# Supplementary material for: Patterns of Intron Gain and Loss in Fungi
Source: PLoS Biol. 2004 Nov 30;2(12):e422. doi: 10.1371/journal.pbio.0020422 (PMC532390; doi:10.1371/journal.pbio.0020422)
Supplement: Table S1 — Also available at http://genes.mit.edu/NielsenEtAl/. (4.3 MB ZIP). [file pbio.0020422.st001.zip › NielsenEtAl/html/192.html]

AN4709.1.NCU00656.1.MG03069.1.FG08522.1


```
 CLUSTAL W (1.82) Multiple Sequence Alignments - Introns Inserted


Sequence 1: NCU00656.1	891 aa
Sequence 2: MG03069.1	970 aa
Sequence 3: FG08522.1	922 aa
Sequence 4: AN4709.1	897 aa
Alignment Length: 1009 aa
Number Identitical Residues: 416 aa
Alignment Score (without introns) 21322


MG03069.1 	---MRETEPFSFGSSDALDFPISVRI~INLEGDEIPYHFSTLLQHPELRHVGSNM-~---
NCU00656.1	---MPIMEPFSFASSDQLNYPVSIRI~MSLEGEETPIPFSTLLERPDLRHVGSNQ-~---
FG08522.1 	MAEYGRMDPFSFAGSKDVHHPVSIRI2MNLEGEEPPFKFSTLLERPDLRHIGSNIR2LTR
AN4709.1  	------MEAFTFAVSAQVDFPIYVKI2GSLEGKQKQIPLSVLLKQPELRHIGSVQ-~---
          	       :.*:*. *  :..*: ::*  .***.:    :*.**::*:***:**       

MG03069.1 	--S~PNSDLYVTVQVWAGSKPLTVPVQTAYKAFKTERR~WNEWLTLPIKYCDVPLNAHLA
NCU00656.1	--S2PHSDLYVTVQVWAGSKPLTVPVQTPYKWFRNERK~WSEWLTLPIDYSTLPENARLA
FG08522.1 	AVS~PNSDLYVTVQVWSGSKPLTVPVQTAYKPFRSERK2WNEWIQLPITYDSLPANSCLA
AN4709.1  	--N~PLSDLFVTAQLWSESKPLGVPLQTSYKAFKTVRA~WNEWLQLPISIKDAPLRCQLA
          	  . * ***:**.*:*: **** **:**.** *:. *  *.**: ***     * .. **

MG03069.1 	ITIWDLSPQPGEGERDQAQGHAVPFGGTTLPLFDGDNQAQKGRQKCHVHRHKLADGCDDT
NCU00656.1	ITLWDLSPTGGEG----AHGHAIPFGGTTLPLFDKDNQLYKGRQQCFVYRHKQADGNDDT
FG08522.1 	ITVWDLSPVGGDE----AIGHAIPFGGTTLPMFDDDNQAQKGRQKCLVHRHKHADGTSNT
AN4709.1  	ITIWDLSPFGGEG----ANGHYIPFGGTTIRLFDDDGKLKTGKQKCKVYRHKAADGFSAT
          	**:*****  *:     * ** :******: :** *.:  .*:*:* *:*** *** . *

MG03069.1 	KTPAILLKRR---NDPRRKDSVVVDEDAEELERMEKLFKKHEMGEIPRVEWLDQLVFRGF
NCU00656.1	GTPAFIPRRSGVGSSKKGTPSSIVDNDAEEYDRLMDLLKKHEMGEIPRVDWLDQLAFRVI
FG08522.1 	KTPALVSTKK---KASRKEQLPPLDKDAEELDRMETLFKKHEMGEIPRVDWLDQMVFRSF
AN4709.1  	TTPSTPSKRR----RGNKPDPLGPSPEELELERVEVLIKKHEMEEIPQIDWMDQLVFRQL
          	 **:    :       .       . :  * :*:  *:***** ***:::*:**:.** :

MG03069.1 	EKRGIQTARSSLKTLQRQRAL---QKANGDDSGSGQEEERASLFKLNIELPRFDFPVVFA
NCU00656.1	EKRGYGSAKSSLRLKAIQRQQ---EALASANPDASKPIPVPSDFLLTVELPRFDFPVVFA
FG08522.1 	EKRGLQAAKSSMKMLQRQQAINGEHDAEGQDGEGKDGRPGQSTFLLNVELPRFDFPVVFA
AN4709.1  	ERLKLSADKAARKRALLMEANR--NRRLESENEEDDEKLEDENFTLYVEFPRFDHPIVWS
          	*:    : ::: :     .     .     :    .     . * * :*:****.*:*::

MG03069.1 	DHGYPPPPISSLQHMSASQSSVALKPPPEVLYGPGIN-SPGEPAGLGTRLVRVYDPEVGA
NCU00656.1	DHEYPPPPISSLQHLSSSQSGLMVKPTPDIQLGPGISRIDADDETSAGRLIRVYDPEVGA
FG08522.1 	DHEYGPPPISALQNLSVSQVSIAHR-QPQVQYGPGINALGEHSTALGGRLIKVYDPEAGY
AN4709.1  	DHEYPPPPISSYPQNAPGNATGTLKSLPEVRFGPGIEGADGE------EVIRIYDPEVGQ
          	** * *****:  : : .:     :. *::  ****.    .      .::::****.* 

MG03069.1 	KDNPAESKHRRLVRGQHRHGVMDKDLKPNPKTRDELNNILSYSPTHPLTPDERDLVWKFR
NCU00656.1	RDNPAESKHRRLVRSQHRNGILDKDLKPNAKVRDELNQIMSYPPTHSLSPEEKDLIWKFR
FG08522.1 	RDNPAEAKHRRLFRSSHRHGILDKDLKPNAKVRDELNLIMSYSPTHVLTPEETDLVWKFR
AN4709.1  	TGNPCEDKHRRLIR-SHRTGIMDRDLKPNPKIRDDLNIIMSYEPTQDLTAEEKDLVWRFR
          	 .**.* *****.* .** *::*:*****.* **:** *:** **: *:.:* **:*:**

MG03069.1 	YHLSRDKRALTKFVKSVSWQDQSESRQAVQVLAKWTEIDVDDALELLGPTFEYPA0L---
NCU00656.1	YHLTRYKKALTKFVKSVNWLDASEARSAVVVLEKWTDIDVDDALELLGPTFSNSA0L---
FG08522.1 	YHLTRDKRALTKFVKSVNWGDQSESRQAIQVLSRWTAIDIDDALELLGPSFDNSA~VRSY
AN4709.1  	YYLTREKKALTKFVKSVNWRDVGEAHQAVEILPKWTEIDVDDALELLGPTFDNAA~VRSY
          	*:*:* *:*********.* * .*::.*: :* :** **:*********:*. .* : : 

MG03069.1 	--------------------VQALKFEHIPTIDRGHREAAEAGGVAAAQDSSLARFLIAR
NCU00656.1	--------------------VQALKYEHILPQSR----------QDVSQDSSLASFLITR
FG08522.1 	AVERLRKADDEELLLYLLQLVQALKYEHISTETE----------EESCQDSSLARFLIQR
AN4709.1  	AVERLRKADDDELLLYLLQLVQALKYEDKAHEDA----------DIAAHDSSLANFLITR
          	: .   .:....      . *****:*.                   .:***** *** *

MG03069.1 	ASQNFMLGNYFYWYLMVECDDLSQDQLPEYRTIYRKVAYDFMTELVARPGGADTRKTLLR
NCU00656.1	AVSSLTLGNYFYWYVMVEIDDRSSEQGQDAREIYTKVAYDFMAELDKQPGGQEKRRTYKR
FG08522.1 	AAANFLLGNYFHWYLMVECDDPSPEQGVDNRNIYRKVAYDFMVELVKQPDGQETRKTLLR
AN4709.1  	AANNFKLGSYLHWYLMVECDDAGPGTLSSHRRLFARVEYYFMAELERMHP--EHRKTLLR
          	*  .: **.*::**:*** ** .     . * :: :* * **.**       : *:*  *

MG03069.1 	QAEWIAILSKISAEIKEANESIARRTDRVKHLLADPKNELITINPPLPLPLDPSQKIVGV
NCU00656.1	QAEWIRILSQVSGEVKEANESIARRTDRVKHFLADSKNELVTLDPPLPLPLDPSVMITGA
FG08522.1 	QAELVAILSRIAQEVKMSNESVAKKVDKVKHFLADPKNELLTFDPPLPMPLDPSILVTGI
AN4709.1  	QGELVAVLSKISKDIRFARENRPIKIEKLKKYLKDPKNDLLQFDPPLPLPLDPDVMVTGC
          	*.* : :**::: ::: :.*. . : :::*: * *.**:*: ::****:****.  :.* 

MG03069.1 	VPDESTVFKSSLHPIKVTFKTDTGQKYPILFKTGDDLRQDQLVVQIITLMDQLLQKENLD
NCU00656.1	IPEETIVFKSSLHPIKVAFKTTSGTKYPLIFKTGDDLRQDQLVIQIIMLMDDLLQKENLD
FG08522.1 	IPDQTIVFKSSLNPFKCTFKTSTGTSYPIIFKLGDDLRQDQLVIQIITLMDQLLQKENLD
AN4709.1  	FPEESNVFKSSLSPLLITFKTTEGRKYPILFKVGDDLRQDQLVIQIIILMDRLLQKENLD
          	.*::: ****** *:  :***  * .**::** **********:*** *** ********

MG03069.1 	LKLSPYKILATSTSAGLTQFVASQSFQTIANRFSRSNVPPALAYLRHHNPDDAAPLGVRK
NCU00656.1	LKLSPYKILATSTSAGLSQFVPSMSFQGILNKYGNN---AALAYLKQNSPDSNGPLGLRK
FG08522.1 	LKLSPYKILATSTSAGASQFVQSASLSSIVNKFKNN---PALAYLKYHNPDDRQPLGVRQ
AN4709.1  	LKLTPYRILATNATAGAMQFIPSTSLSAVSAKYKTV-----LAYLKANNPDENEPLGVRK
          	***:**:****.::**  **: * *:. :  ::        ****: :.**.  ***:*:

MG03069.1 	EAHDMFVRSCAGYCVITYILGVGDRHLDNLLLAPDGHFFHADFGFILGRDPKPFAPAMKL
NCU00656.1	ETLDTFVRSCAGYCVITYILGVGDRHLDNLLLAPDGHFFHADFGYILGRDPKPFAPVMKL
FG08522.1 	ETLDTYVRSCAGYCVITYILGVGDRHLENLLLAPDGHFFHADFGFILGRDPKPFAPLMKL
AN4709.1  	ETMDTYIKSCAGYCVITYLLGVGDRHLENLLLAPDGHFFHADFGFILGRDPKPFAPMMKL
          	*: * :::**********:********:****************:*********** ***

MG03069.1 	SREMVDAMGGAGSDGYRAFKQYCFLAFSALRKSSNLILNLFSLMVDANIPDIKLEPDKAV
NCU00656.1	SKEMVDCMGGVNSEHYRQFKQYCFLAYSALRKNSNLILNLFSLMVDANIPDIKLEPDKAV
FG08522.1 	SKEMVDCMGGVQSEHYKQFKQYCFLSYTALRKSSNLILNLFSLMVDANIPDIRLEPDKAV
AN4709.1  	CKEMVEGMGGTSSPLYLQFKQYCFTAYTTLRKSANLILNLFSLMVDANIPDIRVEPDKAV
          	.:***: ***. *  *  ****** ::::***.:******************::******

MG03069.1 	IKVKDRFHLDMNEEEALRLFERIIDDNLNA2EDYVDLFALRLYGIFTLVKREIYSREYVV
NCU00656.1	FKVRDRFHLELSEEEAIRNLEKIMEDSLN-~-ALVPVMIDRLHGVMQAFRT---------
FG08522.1 	LKVRERFHLELSEEEALRYFDRVIEDTLT-~-AYAPVVIDKLHEWAQAFRA---------
AN4709.1  	LKVKERFHLEMTEEEAIRHFEQLIGDSAN-~-AIFGVVIDRLHDFVQGWRA---------
          	:**::****::.****:* ::::: *. .       :.  :*:      :          

MG03069.1 	DDN1LR0PINTAIRAHLMPGKPGFGGANSPQVRRAEQNSAAKKKRKEPGILRPQYFR
NCU00656.1	---~--~--------------------------------------------------
FG08522.1 	---~--~--------------------------------------------------
AN4709.1  	---~--~--------------------------------------------------
          	
```
